# Deterioration of the Eye

**Published:** 1882-01

**Authors:** 


### DETERIORATION OF THE EYE.

Man progresses—by the law of development—to physical perfection. But by the accidents of civilization the eye, which is the light of the whole body, is in imminent danger of detorioration, and after being evolved by the brute, it is being ruined by man. Already the increase of shortsightedness and color-blindness is attracting considerable attention, and even when these defects are not present the eye of civilization is much inferior to that of many birds and beasts and savages. Not to speak of the cat's ability to see in the dark, what eye can compare for range with that of the condor of the Andes, or for keenness with that of the Indian on the trail of his enemy? Mr. Brudenell Carter, whose address at the Health Congr.ss at Brighton is one of the most interesting and suggestive of recent contributions to popular science, insists upon the importance of checking this gradual deteıioration of the organ of vision. School boards, he says, should educate the eye as well as the tongue, volunteers should institute tests of distant vision, and trade-unions should strike against every employer whose factory is badly lighted. Even the most shortsighted people can see the importance of Mr. Brudenell Carter's warning, and as the spectacle makers are not a very powerful corporation there is some possibility that "science, common sense and humanity" may succeed in arresting the further deterioration of the eye.—*Pall Mall Gazette.*

